# Association between 12‐hr shifts and nursing resource use in an acute hospital: Longitudinal study

**DOI:** 10.1111/jonm.12704

**Published:** 2018-11-21

**Authors:** Peter Griffiths, Chiara Dall'Ora, Nicky Sinden, Jeremy Jones

**Affiliations:** ^1^ National Institute for Health Research Collaboration for Leadership in Applied Health Research and Care (Wessex) University of Southampton Southampton UK; ^2^ Faculty of Health Sciences University of Southampton Southampton UK; ^3^ Corporate Nursing Team Portsmouth Hospitals NHS Trust Hampshire UK

**Keywords:** 12‐hr shifts, health resources, nurses, personnel staffing and scheduling, shift work schedule

## Abstract

**Aim:**

To evaluate whether ≥12‐hr shifts are associated with a decrease in resource use, in terms of care hours per patient day and staffing costs per patient day.

**Background:**

Nurses working long shifts may become less productive and no research has investigated whether potential cost savings are realized.

**Method:**

A retrospective longitudinal study using routinely collected data from 32 wards within an English hospital across 3 years (1 April 2012–31 March 2015). There were 24,005 ward‐days. Hierarchical linear mixed models measured the association between the proportion of ≥12‐hr shifts worked on a ward‐day, care hours per patient day and staffing costs per patient day.

**Results:**

Compared with days with no ≥12‐hr shifts, days with between 50% and 75% ≥12‐hr shifts had more care hours per patient day and higher costs (estimate for care hours per patient day: 0.32; 95% CI: 0.28–0.36; estimate for staffing costs per patient day: £8.86; 95% CI: 7.59–10.12).

**Conclusions:**

We did not find reductions in total care hours and costs associated with the use of ≥12‐hr shifts. The reason why mixed shift patterns are associated with increased cost needs further exploration.

**Implications for Nursing Management:**

Increases in resource use could result in additional costs or loss of productivity for hospitals. Implementation of long shifts should be questioned.

## 1 INTRODUCTION

Traditionally, hospital nursing work was organised around a three‐shift pattern (Josten, Ng, & Thierry, 2003). Often, this consists of an 8‐hr ‘early’ shift commencing at around 7 a.m., followed by an 8‐hr ‘late’ shift from 1.30 p.m. to 9.30 p.m., and a longer ’night’ shift from 9 p.m. to 7 a.m. This pattern necessitates periods where shifts overlap to facilitate handovers. In the case of the early/late shifts, the overlap can be considerable, sometimes up to 2 hr. The move to a two‐shift system, with two long shifts each involving 12 or more hours, began in the late 1970s (Underwood, 1975). Working on a two‐shift system eliminated the long overlap between early and late shifts, offering potential efficiency savings without compromising the nurse‐to‐patient ratio available throughout the day (Ganong, Ganong, & Harrison, 1976). As nurse‐to‐patient ratios are widely acknowledged as important for patient safety (Aiken et al., 2014; Ball et al., 2018; Griffiths et al., 2016), it appears that safety could be maintained with fewer total care hours.

Despite this, the use of 12‐hr shifts to organise the delivery of nursing services in acute hospitals remains controversial. Studies have found longer nursing shifts to be associated with decreased job satisfaction, increased burnout, worse nurse reported care quality and increased mortality at a hospital level (Dall'Ora, Griffiths, Ball, Simon, & Aiken, 2015; Griffiths et al., 2014; Rogers, Hwang, Scott, Aiken, & Dinges, 2004; Scott, Rogers, Hwang, & Zhang, 2006; Stimpfel, Lake, Barton, Gorman, & Aiken, 2013; Stimpfel, Sloane, & Aiken, 2012; Trinkoff et al., 2011). Nurses working 12‐hr shifts report higher levels of necessary care being left undone due to lack of time (Ball et al., 2017; Griffiths et al., 2014).

This evidence provides sufficient cause to re‐examine the move to 12‐hr shifts. While it appears self‐evident that the revised shift patterns will reduce total care hours, evidence indicating that 12‐hr shifts are associated with increased rates of nursing care being delayed or omitted raises the possibility that nurses may become less efficient, undermining the often claimed productivity gains (Griffiths et al., 2014). If that was the case, the total hours of care required to meet patient need may need to increase in order to compensate. The widespread assumption of a net reduction in staff costs has never been tested. In this paper we address one of the fundamental motivators for the adoption of 12‐hr shifts—the assumption that it provides a more efficient use of resources by reducing the number of paid hours worked by registered nurses and health care assistants (HCAs). We aimed to determine how the total hours of care and associated staffing costs change with variation in the use of 12‐hr shifts within and between wards over time.

## 2 METHODS

We performed a retrospective observational study using routinely collected data on nursing staff shifts in all inpatient general adult wards (*n *=* *32) in a large acute care hospital Trust in the South of England. The University of Southampton Ethics Committee granted ethical approval to undertake this research (submission number 18311). Data were drawn from a large parent study (ISRCTN registration 17930973 http://www.isrctn.com/ISRCTN17930973).

### 2.1 Data sources

All shifts worked between 1 April 2012 and 31 March 2015 by substantive nursing staff in the study wards were extracted from a hospital‐wide electronic system (i.e., E‐Roster). Each shift record included worked hours (shift date, start and end time), ward, and nursing staff grade. A second database recorded all bank (i.e., extra contractual work within the Trust by staff employed by the Trust) and agency shifts (i.e., shifts worked by staff employed through an external agency). Shifts with codes indicating absence were removed prior to calculating ward staffing levels and we included ward based shifts only. Patient data were extracted from patient administration datasets. These data included National Early Warning Score (NEWS) of patients. Data from the hospital's Patient Administration System were used to determine the number of patients on each ward‐day.

### 2.2 Study variables

The outcome was resource use, captured by two variables: care hours per patient day (CHPPD) and staffing costs per patient day. The NHS nursing workforce is composed of registered nurses (RNs) and HCAs, also known as health care support workers. For each ward, care hours for each day (i.e., from midnight to midnight) were calculated by summing all worked hours between the shifts’ start time and end time, removing time allocated for breaks. Shifts longer than 11 hr are associated with a 1‐hr unpaid break; shifts shorter than 11 hr are associated with a 30 min unpaid break.

The number of patient days for each ward was calculated using the admission, discharge and transfer information over a 24‐hr period (i.e., midnight to midnight). For example, a patient occupying the bed for 12 hr would be assigned a value of 0.5 (patient hours/24 hr). Consequently, patient days represents the average number of occupied beds in a 24‐hr period.

Nurse staffing costs per patient day were calculated using the Unit Costs of Health and Social Care (Curtis & Burns, 2016) by including salary costs only. In order to account for changes in cost arising from wage inflation and changes in taxation, 2015 costs were applied to all years. For registered nurse staffing we calculated a hospital weighted average of the unit costs of band 5, 6 and 7. For health care assistant staffing, we calculated a hospital weighted average of the unit costs of band 2, 3 and 4. For each ward‐day, we calculated the proportion of CHPPD deriving from ≥12‐hr shifts. We excluded ward‐days that were one or more CHPPD below/above the 1st/99th centile, as these were extreme outliers, likely indicating invalid data.

To account for a positive association between patient acuity and required staffing levels (Twigg & Duffield, 2009), we calculated the proportion of unwell patients in each ward‐day, defined as the proportion of patients that had a National Early Warning Score (NEWS) of three or above. A NEWS score of three defines a patient as at medium risk of deterioration, requiring a minimum of 4‐hourly vital signs observations (Royal College of Physicians, 2012).

### 2.3 Statistical analysis

First, we produced descriptive reports of distributions and frequencies of the proportion of ≥12‐hr shifts, CHPPD and nurse staffing costs per patient day, taking into account variation across wards and over time (in 6‐month intervals). In order to perform descriptive and multilevel regression analysis, the proportion of CHPPD deriving from ≥12‐hr shifts was grouped into five categories: 0; >0 to ≤0.25; >0.25 to ≤0.50; >0.50 to ≤0.75; >0.75. We then measured the unadjusted relationship between the proportion of ≥12‐hr shifts and total CHPPD and nurse staffing costs per patient day, by calculating the Pearson correlation coefficient and producing a table of means for each category.

Finally, we used linear mixed models to explore the relationship between the use of ≥12‐hr shifts and resource use. The proportion of unwell patients was added as a control variable and ward was added as a random effect. We performed a sub‐group analysis of wards which changed from having a low proportion of ≥12‐hr shift patterns at the beginning of the study (≤20%), but which had moved to a majority of ≥12‐hr shift patterns by the end of the study (≥60%). All analyses were performed using R (R Development Core Team, 2017) and the package lme4 for linear mixed models (Bates, Mächler, Bolker, & Walker, 2015).

## 3 RESULTS

The analytic sample consisted of 24,005 ward‐days. Across the 3 years, on average 47% of CHPPD derived from ≥12‐hr shifts, with large variation (interquartile range = 0%–80%). There was an average of 7.03 CHPPD. The mean staffing cost per patient day was £224.20 ($310.95; €251.22^1^ ) (Table 1). For a detailed description of ward characteristics, please see Appendix 1.

**Table 1:** Mean, median and interquartile range of study variables

	Mean	Median	Interquartile range (IQR)	Min	Max
Proportion of ≥12‐hr shifts	0.47	0.61	0–0.80	0	1
CHPPD	7.03	6.76	5.78–7.85	2.41	16.40
Cost per patient day (£)	224.20	211.80	180.10–252.30	81.20	552.30

**Figure 1:**
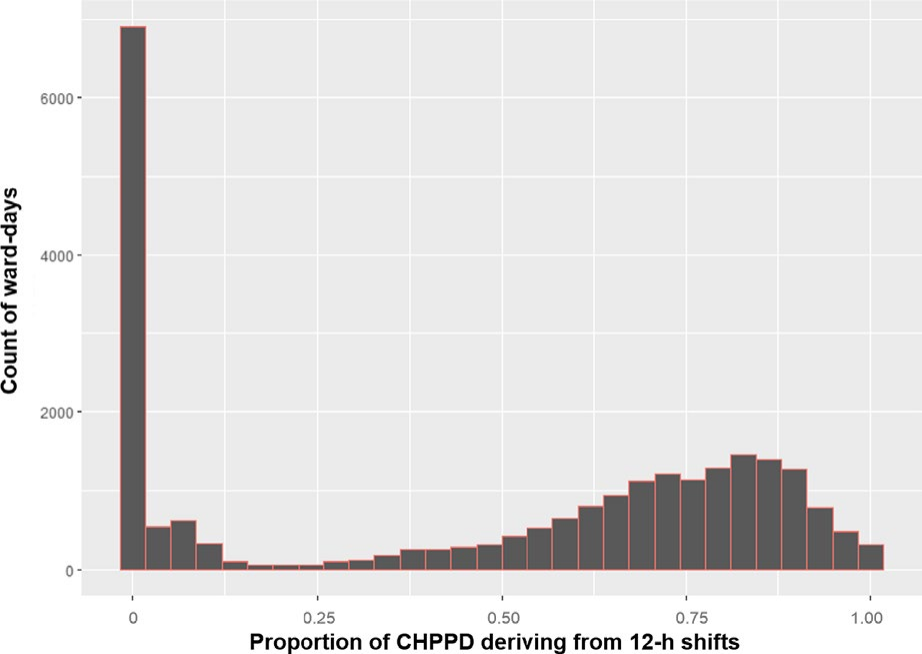
Histogram of distribution of proportion of CHPPD deriving from 12‐hr shifts by ward‐days [Colour figure can be viewed at wileyonlinelibrary.com]

In the largest group of ward‐days (*n *=* *6,837, 28.4%), no ≥12‐hr shifts were worked (i.e., 0% of CHPPD were derived from ≥12‐hr shifts). In 311 ward‐days (1.2%) all CHPPD were fulfilled by ≥12‐hr shifts (Figure 1). The median proportion of CHPPD deriving from 12‐hr shifts was 0.61. The distribution of proportions of ≥12‐hr shifts was explored by ward at two different points in time, at the first 6 months of the study (i.e., April 2012–September 2012) and at the last 6 months of the study (October 2014–March 2015). There was a substantial move towards ≥12‐hr shifts during the course of the study. Across all wards the median proportion of ≥12‐hr shifts in the first 6 months of the study was 0.04, indicating that for the majority of wards fewer than 5% of hours worked derived from ≥12‐hr shifts. In the last 6 months of the study, the median for the proportion of ≥12‐hr shifts was 0.71, indicating that the majority of hours worked were derived from ≥12‐hr shifts in a majority of wards.

There was a weak positive correlation between the total CHPPD and the proportion of hours derived from ≥12‐hr shifts (*r *=* *0.10) and a weak positive correlation between staffing cost per patient day and the proportion of hours derived from ≥12‐hr shifts (*r *=* *0.14).

We explored average CHPPD and average staffing cost per patient day by categories of proportions of CHPPD deriving from ≥12‐hr shifts (Table 2). Compared with days when no hours were derived from ≥12‐hr shifts, the average CHPPD were slightly lower when up to half CHPPD derived from ≥12‐hr shifts, but increased when the proportions of CHPPD deriving from ≥12‐hr shifts were higher than 0.50. Similarly, staffing costs increased when more than half of CHPPD were deriving from ≥12‐hr shifts.

**Table 2:** Average CHPPD and staffing costs by proportion of ≥12‐hr shifts

Proportions of CHPPD from ≥12‐hr shifts	Ward‐days	Average CHPPD	Average nurse staffing cost per patient day (£)
0	6,837	6.81	215
0 to ≤0.25	1833	6.66	207.6
0.25 to ≤0.50	1,510	6.40	202.7
0.50 to ≤0.75	5,950	7.28	232.9
0.75	7,875	7.16	233.5

Because these univariate associations do not account for ward level effects or possible changes in staffing requirements due to changes in patient acuity, we explored these relationships with linear mixed models, controlling for the proportion of unwell patients, and with ward as a random effect. The results are summarized in Table 3.

**Table 3:** Outputs of linear mixed models of the association between proportion of CHPPD deriving from ≥12‐hr shifts and total CHPPD and staffing costs per patient day

	CHPPD		Staffing costs per patient day (£)	
	Estimate	95% CI	Estimate	95% CI
Proportion of CHPPD deriving from ≥12‐hr shifts (quartiles)				
0 (reference category)				
>0 to ≤0.25	0.23^a^	0.18–0.29	6.79^a^	5.13–8.45
>0.25 to ≤0.50	0.22^a^	0.14–0.29	7.14^a^	4.79–9.48
>0.50 to ≤0.75	0.32^a^	0.28–0.36	8.86^a^	7.59–10.12
>0.75	0.06^a^	0.01–0.11	−0.38	−1.26–2.04
Proportion of unwell patients	0.24^a^	0.10–0.39	8.63^a^	4.01–13.26

Notes

All models included Ward as random effects. Total sample: 24,005 ward‐days.

a Significant at *p *<* *0.05.

When the proportion of CHPPD from ≥12‐hr shifts was higher than 0 (that is, when any ≥12‐hr shifts were worked) the total CHPPD were significantly higher (*p *<* *0.05). The strongest effect was observed when ≥12‐hr shifts accounted for more than 50% but less than 75% CHPPD. Estimates of staffing costs per patient day increased with the proportion of CHPPD from ≥12‐hr shifts up to 0.75. Although costs were marginally (<£1 per patient per day) decreased when the proportion of hours from 12‐hr shifts was >0.75, the association was not significant.

In order to further control for possible ward level confounding, we undertook a sub‐group analysis including only wards that changed from using predominately 8‐hr shifts to ≥12‐hr shifts during the course of the study. Overall, 14 wards were in this subgroup. For these wards, the median proportion of CHPPD deriving from ≥12‐hr shifts was 0 in the study first 6 months (1 April 2012–30 September 2012). In the last 6 months (1 October 2014–31 March 2015), the median was 0.64, indicating a substantial shift to longer hours across the 3 study years. Linear mixed models were fitted for this subgroup, and the results can be found in Table 4.

**Table 4:** Outputs of linear mixed models of the association between proportion of CHPPD deriving from 12‐hr shifts and total CHPPD and staffing costs per patient day in the changers subgroup

	CHPPD		Staffing costs per patient day (£)	
	Estimate	95% CI	Estimate	95% CI
Proportion of CHPPD deriving from ≥12‐hr shifts (quartiles)				
0 (reference category)				
>0 to ≤0.25	0.27^a^	0.22–0.33	7.37^a^	5.60–9.15
>0.25 to ≤0.50	0.36^a^	0.26–0.45	11.47^a^	8.54–14.40
>0.50 to ≤0.75	0.36^a^	0.31–0.40	10.58^a^	9.19–11.98
>0.75	0.07	0.00–0.14	0.90	−1.55 to 3.35
Proportion of unwell patients	−0.18	−0.39 to 0.03	−4.66	−11.45 to 2.17

All models included Ward as random effects. Total sample: 24,005 ward‐days.

a Significant at *p *<* *0.05.

The sub‐group analysis provided similar results to the overall analysis, but the increased resource use associated with high proportions of long shifts was larger. When the proportion of hours derived from ≥12‐hr shifts was >0.50 to ≤0.75 the cost per patient per day was increased by £10.58 and when proportion of hours derived from ≥12‐hr shifts was >0.25 to ≤0.50, the cost per patient per day was increased by £11.47, compared with ward‐days where no ≥12‐hr shifts were worked.

## 4 DISCUSSION

This was the first study to analyse whether the increased use of shifts of 12 hr or longer is associated with a decrease in resource use. When we controlled for ward level effects and patient acuity, we found that days where some hours were worked as 12‐hr shifts were associated with small but statistically significant increases in total CHPPD and costs, until the proportion of hours derived from 12‐hr shifts exceeded 0.75 (75%). Above this level, total CHPPD and staff costs did not differ significantly from when no 12‐hr shifts were worked. Our sub‐group analysis of wards that changed from low to high use of 12‐hr shifts confirmed this pattern. We found no evidence that increased use of 12‐hr shifts was associated with decreases in CHPPD or costs.

In some countries such as the USA and Ireland, 12‐hr shifts are the norm, whereas they remain a rarity in many European countries (Griffiths et al., 2014). England has previously been identified as in transition with 30% of nurses reporting working 12‐hr shifts in 2011 (Griffiths et al., 2014) increasing to 50% in 2017 (Royal College of Nursing, 2017). In the hospital we studied, in 2012 on average 4% of the CHPPD worked were part of ≥12‐hr shifts whereas in 2015, on average 71% of CHPPD came from ≥12‐hr shifts. Nonetheless, although the prevalence of 12‐hr shifts dramatically increased, the predominant pattern was one of wards using a mixed pattern of shifts.

The literature on 12‐hr shifts has frequently cited increased productivity and efficiency as a motivation for moving from an 8‐hr shift system (Thomson, Schneider, & Hare Duke, 2017). Claims are made on the basis of a reduction in shift overlaps and the number of handovers required. While these claims have been reiterated, we have not been able to find any robust empirical literature quantifying the claims, although a single short case report from the NHS claims savings of up to 14% in nursing hours based on projections from a single case study (NHS Evidence, 2010). It is not clear if this estimate was based on a theoretical projection or observational data. Our findings suggest that the theoretical claims may not be realized in practice.

It has long been recognized that long shifts might be associated with reduced productivity due to the need of nurses to pace themselves during shifts (Reid, Robinson, & Todd, 1993). There is some evidence to support this as nurses working 12‐hr shifts were more likely to report leaving necessary care undone (Griffiths et al., 2014) and lower productivity was also reported by some nurses working in the NHS case study cited above. It may be that this reduced productivity leads to an increased demand for staff to properly meet patient need, thus negating any initial savings. The initial savings may never be realized if a mixture of shift patterns is used since the mixed pattern potentially increases rather than reduces the number of handovers. The relative improvements in total CHPPD and costs once more than 75% of hours are worked as 12‐hr shifts, may result from a reduction in handovers, although resource use never falls below that observed when no 12‐hr shifts are worked.

The increased costs per patient per day are relatively modest and might be justifiable if they were resulting in improvements in quality and safety of care, or other tangible benefits with no risk. A recent literature review found that evidence of benefits beyond nurse preferences is elusive, with findings suggesting adverse outcomes for both nurses and patients (Dall'Ora, Ball, Recio‐Saucedo, & Griffiths, 2016). A report based on the data used in the present study found that the odds of missing a 12‐hr shift due to sickness absence were 24% higher than for an 8‐hr shift. Furthermore, when nurses had worked more than 75% of their shifts as ≥12‐hr shifts over the past week, their odds of experiencing both short‐term and long‐term sickness absence were significantly increased (Dall'Ora et al., 2018). NHS staff sickness absence is both costly and has negative consequences on patient care (Duclay, Hardouin, Sebille, Anthoine, & Moret, 2015; The Health Foundation, 2015). The negative impact of long shifts on job satisfaction and intention to leave has been reported by a recent European study (Dall'Ora et al., 2015).

This study has some limitations: due to the single‐site nature of the study, there should be a cautious approach to generalization. Each hospital in England is different, but there tends to be more variation in shift patterns within hospitals than there is between hospitals (Griffiths et al., 2014). While the mixed shift patterns that we observed may or may not be common in other hospitals, we found no evidence for a reduction in costs or CHPPD when a high proportion of 12‐hr shifts were used. It is also possible that variation in CHPPD and staffing costs were due to factors which could not be captured in our research, although patient acuity could be controlled for. We were not able to take into consideration nurse demographic information, including age, length of service in the hospital, years of experience. However, because we have costed RN and HCAs hours based on grade, we are confident that the attributed costs are accurate.

A full economic analysis is needed to determine the costs and consequences of moving to 12‐hr shifts, especially considering the effects of such shift patterns on staff sickness absence, although other evidence does not make it seem likely that there would be resource use benefits arising from improved patient outcomes.

While reductions in the total care hours required is an often cited benefit of the use of 12‐hr shifts, it is not the only motivation and it was not necessarily a goal of this hospital. Also, our study was observational and increases in resource use associated with increases in the use of 12‐hr shifts should not be interpreted as direct evidence of cause. Nonetheless, since efficiency savings associated with the move to 12‐hr shifts are often assumed to be axiomatic, our failure to observe any evidence for such reductions is striking.

## 5 CONCLUSIONS

The increasingly common move to work patterns including 12‐hr shifts based, in part, on presumed savings on staffing and a more cost‐effective resource use should be questioned. Our findings suggest that such savings are not achieved. While there was no net increase in costs or resource use when a high proportion of hours were derived from 12‐hr shifts, there were no increases and other evidence suggests that increased costs are more likely than savings. The use of mixed patterns of 12 and 8‐hr shifts appears to be particularly resource intensive although the impact of these patterns on patient outcomes are less well studied.

## 6 IMPLICATIONS FOR NURSING MANAGEMENT

A major objective for nurse managers is to deploy the nursing workforce so that good quality of patient care can be achieved, while avoiding excessive spending. As this research has found, how shift patterns are organised has implications for nursing resource use, in terms of CHPPD and nurse staffing costs. These findings suggest that deploying mixed shift patterns may lead to higher resource use. Therefore, nurse managers should question the routine implementation of long shift patterns, especially where this is based on assumed cost savings.

## ETHICAL APPROVAL

An ethics application was submitted to the University of Southampton's ethics committee through ERGO and was granted approval by the Research Governance Office (Submission Number 18311).

## Note

1 Currencies converted using OANDA currency converter on 07/03/2018 https://www.oanda.com/currency/converter/.
