# Supplementary material for: Association between 12‐hr shifts and nursing resource use in an acute hospital: Longitudinal study
Source: J Nurs Manag. 2018 Nov 21;27(3):502–8. doi: 10.1111/jonm.12704 (PMC7380133; doi:10.1111/jonm.12704)
Supplement: Supplementary file 1 [file JONM-27-502-s001.docx]

Appendix 1 – CHPPD, number of nursing staff and number of beds by ward

| Ward | Days of Staffing Data* | Number of beds | Number of Nursing Staff | CHPPD  Mean (SD) | Skill Mix  Mean  (SD) |
| --- | --- | --- | --- | --- | --- |
| Oncology | 1054 | 40 | 109 | 7.2 (0.8) | 0.71 (0.02) |
| Medical – Gastro | 1044 | 36 | 72 | 6.4 (1) | 0.50 (0.07) |
| Coronary Care | 1071 | 23 | 48 | 7.2 (0.8) | 0.83 (0.06) |
| Cardiology | 1028 | 36 | 62 | 5.6 (0.7) | 0.51 (0.05) |
| Trauma 1 | 691 | 28 | 59 | 7.3 (1.1) | 0.54 (0.06) |
| General Medicine 1 | 427 | 30 | 47 | 6.5 (1) | 0.45 (0.05) |
| General Medicine 2 | 906 | 34 | 58 | 5.5 (0.8) | 0.58 (0.05) |
| Trauma 2 | 621 | 26 | 55 | 6.8 (0.8) | 0.55 (0.05) |
| Orthopaedics 1 | 479 | 36 | 46 | 6.4 (1.2) | 0.56 (0.05) |
| Orthopaedics 2 | 523 | 36 | 62 | 7.4 (1.2) | 0.52 (0.05) |
| Orthopaedics 3 | 163 | 13 | 31 | 8.1 (1.3) | 0.56 (0.06) |
| Surgical 1 | 818 | 30 | 56 | 5.2 (0.5) | 0.60 (0.05) |
| Surgical 2 | 897 | 32 | 61 | 5.4 (0.7) | 0.59 (0.05) |
| Respiratory 1 | 1092 | 36 | 60 | 5.4 (0.8) | 0.50 (0.07) |
| Respiratory 2 | 854 | 40 | 83 | 7.1 (0.6) | 0.67 (0.04) |
| Medicine for older people 1 | 930 | 13 | 79 | 7.7 (1.4) | 0.50 (0.06) |
| Medicine for older people 2 | 140 | 30 | 25 | 7.3 (0.9) | 0.46 (0.05) |
| Medicine for older people 3 | 1011 | 23 | 73 | 6.5 (0.6) | 0.52 (0.04) |
| Medicine for older people 4 | 951 | 29 | 84 | 6.9 (0.8) | 0.52 (0.05) |
| Medicine for older people 5 | 757 | 30 | 49 | 7.6 (0.9) | 0.47 (0.08) |
| Stroke Unit 1 | 374 | 25 | 62 | 7 (0.5) | 0.46 (0.05) |
| Stroke Unit 2 | 942 | 34 | 85 | 6.7 (0.8) | 0.53 (0.06) |
| Renal 1 | 868 | 10 | 47 | 11.2 (1.6) | 0.78 (0.06) |
| Renal 2 | 740 | 26 | 56 | 8.4 (1) | 0.79 (0.05) |
| Renal 3 | 323 | 14 | 31 | 8.6 (1.2) | 0.75 (0.05) |
| Gynaecology | 334 | 22 | 46 | 7.9 (1.5) | 0.66 (0.07) |
| Head & Neck | 724 | 27 | 51 | 6.3 (1.2) | 0.68 (0.07) |
| Medical Assessment Unit | 1088 | 58 | 155 | 8.9 (1.3) | 0.71 (0.03) |
| Private Patients Unit | 267 | 13 | 30 | 8.3 (1.8) | 0.69 (0.05) |
| Surgical Assessment Unit | 892 | 28 | 53 | 5.2 (0.9) | 0.65 (0.06) |
| Surgical High Intensity Unit | 1075 | 10 | 31 | 10 (1.5) | 0.79 (0.06) |
| Urology | 921 | 31 | 78 | 5.6 (0.7) | 0.55 (0.04) |
| * Wards have different number of days due to ward closures; wards opening later during the study; ward-days removed due to invalid data | | | | | |
|  | | | | | |
